# Gene expression profile analysis of colon cancer grade II into grade III transition by using system biology 

**Published:** 2019

**Authors:** Mohammad Rostami-Nejad, Sina Rezaei Tavirani, Vahid Mansouri, Somayeh Jahani-Sherafat, Hamideh Moravvej Farshi

**Affiliations:** 1 *Gastroenterology and Liver Diseases Research Center, Research Institute for Gastroenterology and Liver Diseases, Shahid Beheshti University of Medical Sciences, Tehran, Iran *; 2 *Proteomics Research Center, Faculty of Paramedical Sciences, Shahid Beheshti University of Medical Sciences, Tehran, Iran *; 3 *Microbiology Department, Faculty of Medicine, Isfahan University of Medical Sciences, Isfahan, Iran*; 4 *Skin Research Center, Shahid Beheshti University of Medical Sciences, Tehran, Iran *

**Keywords:** Colon cancer, gene, biomarker

## Abstract

**Aim:**

Gene expression profile analysis of colon cancer grade II into grade III transition by using system biology.

**Background:**

Colon cancer is one of lethal cancer in men and women. Treatment in advanced colon cancer is difficult and survival rate is low.

**Methods:**

Gene expression profiles of children patients with non-preforated appendicitis in comparison with the samples with non- appendicitis abdominal pain are analysis via protein – protein interaction PPI and the critical compounds are introduced by STITCH.

**Results:**

Six critical genes including MAPK3, AKT1, SRC, TP53, GAPDH, and ALB were identified as a possible biomarker panel related to colon cancer grade II to III transition. Among these critical genes roles of MAPK3, AKT1, SRC, TP53 are highlighted.

**Conclusion:**

It was concluded that target therapy to regulate SRC and TP53 may be the effective therapeutic way to treatment of colon cancer and more researches in necessary to design drugs for these purposes.

## Introduction

 Colon cancer (CC) is the third common cases of cancers in the world with near 5-year survival of 50% (1).Colorectal cancers could be graded due to the size of the tumor and the amount of invasion as well as the rate of tumor node metastasis (TNM) stages (2). American joint committee on cancer endorses TNM stages I to IV for CC. Stages I to III have no invasion to the serosa, treated by surgery and sometimes chemotherapy but in stage IV with metastasis, chemotherapy is obligatory (1). Since the rate of disease recurrence is not specified in stages 1 to 4, surgery resection is used, but the disease may recur again 3 years after surgery (3). Therefore, early diagnosis of the disease can take place at the stages of treatment but there is currently no method to detect early stages of CC. Development of effective biomarkers to detect CC is essential to improve therapeutic methods. Proteomics can be a promising proposition for intercepting colon cancer-related bio-markers. Especially, blood biomarkers are useful in proteomics examinations. Recently, using databases, it is possible to aggregate and analyze the existing data on colorectal cancers (4). Through the information obtained from these databases, it is possible to draw up relevant protein-protien interaction networks (5).

Over the past 10 years, omics studies has introduced many candidates as biomarkers for cancer disease but there is no evidence for effective biomarker involved in CC(6-8). Takashi Shiromizu et al introduced the sensitivities of annexins A3, A4, and A11 peptides for detecting early-stage colorectal cancer and they believed these few peptides as promising biomarkers for early detection of disease (9).

**Table 1:** Critical nodes of colon cancer grades II and III network. BC and CC refer to betweenness centrality and closeness respectively

**R**	**Display name**	**description**	**Degree**	**BC**	**CC**
1	GAPDH	glyceraldehyde-3-phosphate dehydrogenase	134	0.032878	0.671916
2	AKT1	v-akt murine thymoma viral oncogene homolog 1	123	0.024122	0.644836
3	ALB	albumin	122	0.027529	0.644836
4	TP53	tumor protein p53	119	0.038235	0.636816
5	SRC	v-src sarcoma (Schmidt-Ruppin A-2) viral oncogene homolog (avian)	118	0.025134	0.636816
6	MAPK3	mitogen-activated protein kinase 3	118	0.020113	0.635236

**Table 2:** 34 biological terms related to the network of colon cancer grades II and III. % G/T and G/T refer to percentage of genes per term and contribution of numbers of genes in term. The rows 1- 16, 17, 18 – 33, and 34 are retrieved from KEGG_20.11.2017, REACTOME_Pathways_20.11.2017, WikiPathways_20.11.2017, and GO_BiologicalProcess-EBI-QuickGO GOA_20.11.2017_00h00 respectively

**R**	**GOTerm**	**% G/T**	**G/T**	**Associated Genes Found**
1	HIF-1 signaling pathway	3.00	3	[AKT1, GAPDH, APK3]
2	ErbB signaling pathway	3.49	3	[AKT1, MAPK3, SRC]
3	VEGF signaling pathway	5.08	3	[AKT1, MAPK3, SRC]
4	Estrogen signaling pathway	3.06	3	[AKT1, MAPK3, SRC]
5	Prolactin signaling pathway	4.29	3	[AKT1, MAPK3, SRC]
6	Thyroid hormone signaling pathway	3.45	4	[AKT1, MAPK3, SRC, TP53]
7	Colorectal cancer	4.17	3	[AKT1, MAPK3, TP53]
8	Pancreatic cancer	4.00	3	[AKT1, MAPK3, TP53]
9	Endometrial cancer	5.17	3	[AKT1, MAPK3, TP53]
10	Glioma	4.23	3	[AKT1, MAPK3, TP53]
11	Prostate cancer	3.09	3	[AKT1, MAPK3, TP53]
12	Melanoma	3.95	3	[AKT1, MAPK3, TP53]
13	Bladder cancer	7.32	3	[MAPK3, SRC, TP53]
14	Chronic myeloid leukemia	3.85	3	[AKT1, MAPK3, TP53]
15	Non-small cell lung cancer	4.55	3	[AKT1, MAPK3, TP53]
16	Central carbon metabolism in cancer	4.62	3	[AKT1, MAPK3, TP53]
17	PI5P, PP2A and IER3 Regulate PI3K/AKT Signaling	3.19	3	[AKT1, MAPK3, SRC]
18	RANKL/RANK (Receptor activator of NFKB (ligand)) Signaling Pathway	5.45	3	[AKT1, MAPK3, SRC]
19	Human Thyroid Stimulating Hormone (TSH) signaling pathway	4.55	3	[AKT1, MAPK3, SRC]
20	Leptin signaling pathway	3.95	3	[AKT1, MAPK3, SRC]
21	Follicle Stimulating Hormone (18) signaling pathway	11.11	3	[AKT1, MAPK3, SRC]
22	Prolactin Signaling Pathway	3.95	3	[AKT1, MAPK3, SRC]
23	Signaling Pathways in Glioblastoma	4.82	4	[AKT1, MAPK3, SRC, TP53]
24	AGE/RAGE pathway	4.55	3	[AKT1, MAPK3, SRC]
25	Interleukin-11 Signaling Pathway	6.82	3	[AKT1, MAPK3, SRC]
26	Oncostatin M Signaling Pathway	6.15	4	[AKT1, MAPK3, SRC, TP53]
27	Alpha 6 Beta 4 signaling pathway	9.09	3	[AKT1, MAPK3, SRC]
28	IL-3 Signaling Pathway	6.12	3	[AKT1, MAPK3, SRC]
29	Kit receptor signaling pathway	5.08	3	[AKT1, MAPK3, SRC]
30	Rac1/Pak1/p38/MMP-2 pathway	5.97	4	[AKT1, MAPK3, SRC, TP53]
31	Hepatitis C and Hepatocellular Carcinoma	5.88	3	[AKT1, MAPK3, TP53]
32	Endometrial cancer	5.17	3	[AKT1, MAPK3, TP53]
33	EPO Receptor Signaling	11.54	3	[AKT1, MAPK3, SRC]
34	regulation of telomerase activity	5.88	3	[MAPK3, SRC, TP53]

Kit et al represented protein kinase C gamma, c-Myc, MDM2, bCOP, p14ARF, p57kip2, GRB2, APP, pan cytokeratin, up regulated in CC (10). Liang B Liang et al identified top 10 hub genes, GNG2, AGT, SAA1, ADCY5, LPAR1, NMU, IL8, CXCL12, GNAI1, and CCR2 from the PPI network using bioinformatics software (11). As it is evident, different biomarkers have been introduced in different metabolic pathways by different scholars and all researchers are trying to find key biomarkers for colon cancer. The use of databases has recently become useful in this regard. In this study, we aim to compare the colon cancer II & III genome in two groups of patients to find the metabolic pathways for the important genes and proteins involved in these two grades. 

## Methods

Gene expression profiles of 3 colon cancer patients grade II and 3 individuals with colon cancer grade III were obtained from GEO (GSE25071, GPL/2986). Age of colon cancer grade II male patients was 36, 46, and 49 years. The male patients with grade III colon cancer were 43, 46, and 47 years old. Numbers of top significant 250 DEGs were selected to further analysis. Fold changes less than 0.5 and more than 2 were considered. The uncharacterized DEGs were deleted and the remaind ones included to PPI network analysis by Cytoscape software v 3.6.0 (12) and its application STRING database (13). The network was analyzed by Network Analyzer a plugin of Cytoscape to find the central nodes. Cutoff mean +2SD was considered to determining hub nodes (14). Top 5% of nodes based on betweenness centrality and closeness centrality were selected as central nodes (15). Common nodes between central nodes and hubs were introduced as critical DEGs. Action map including activation, inhibition, binding, and expression related to the 6 critical DEGs was constructed by CluePedia (16) application of Cytoscape. Gene ontology enrichment of 6 critical nodes was done be ClueGO (17) to determined biological pathways and the other biological terms. In all analysis p-value less than 0.05 was regarded. 

## Results

**Figure 1:**
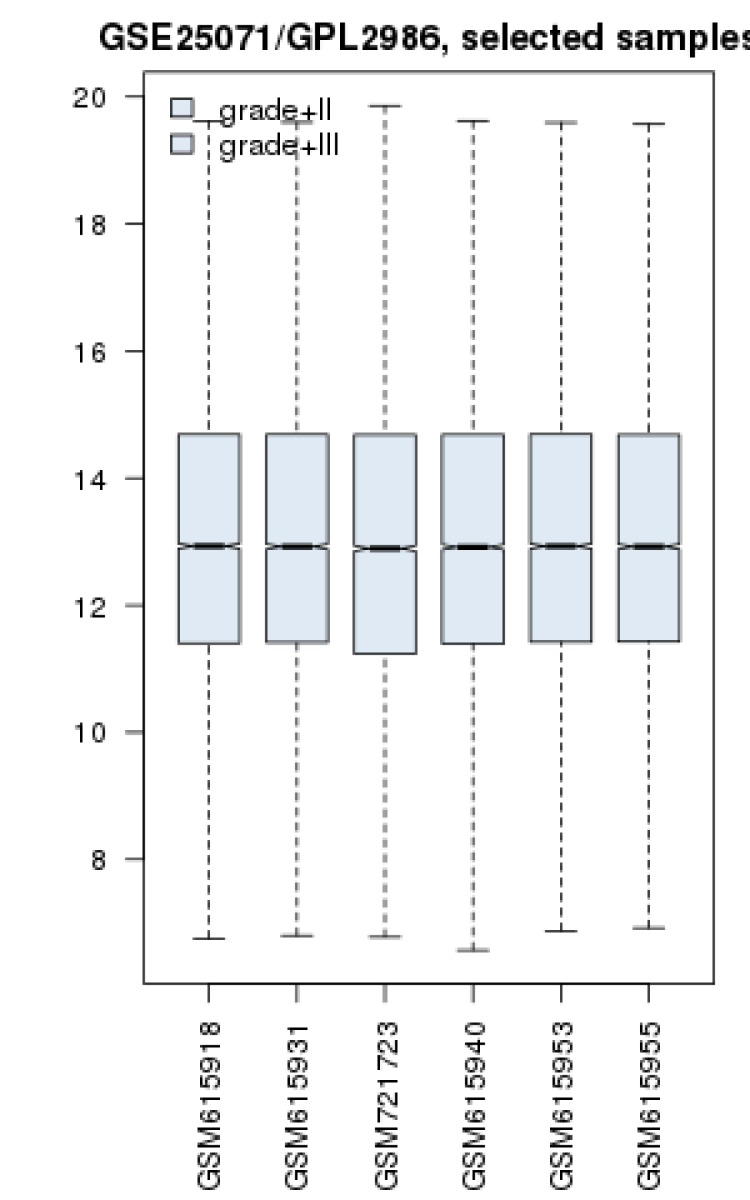
Box plot presentation of three gene profiles of colon cancer grade II samples (GSM615918, 31, and GSM721723) and grade III (GSM 615940, 53, and 55)

**Figure 2:**
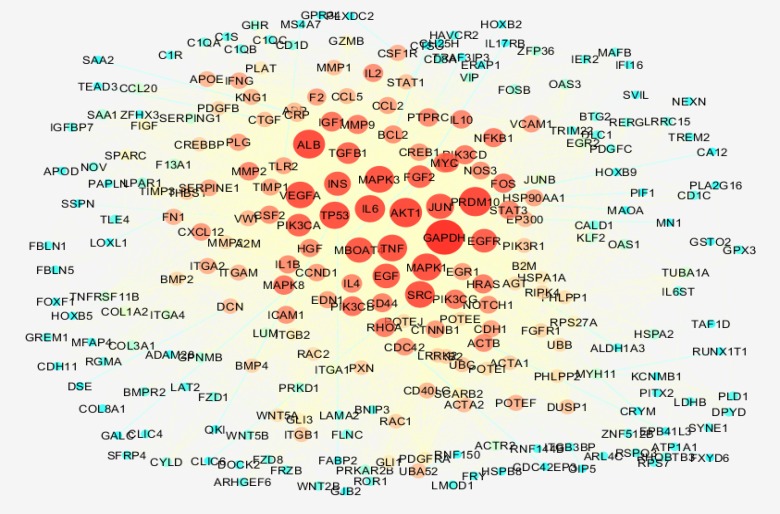
Main connected component of colon cancer grades II and III. The nodes are layout based on degree value. Color from blue to red refers to increment of degree

Gene profiles of colon cancer grades II and III were matched via box plot analysis (see figure 1). The samples are median centered and are comparable. Fold change of all top significant 250 DEGs were above 2 and less than 0.5. Numbers of 195 individual among 250 DEGs were characterized and except one DEGS, all of them were recognized by STRING database. The network was constructed by 194 DEGs and 100 added relevant genes (see figure 2).

**Figure 3:**
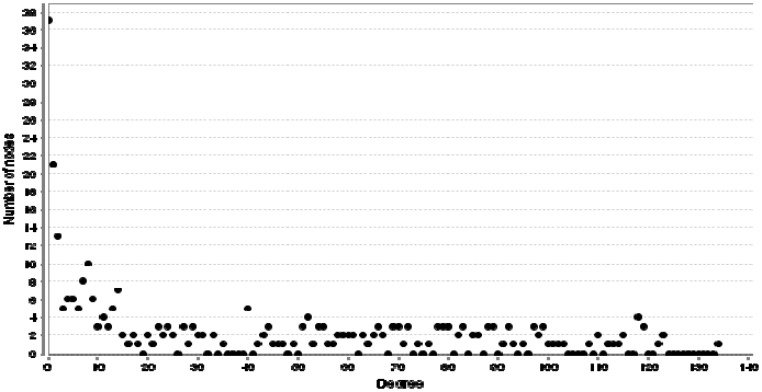
Degree distribution of colon cancer grades II and III network

**Figure 4:**
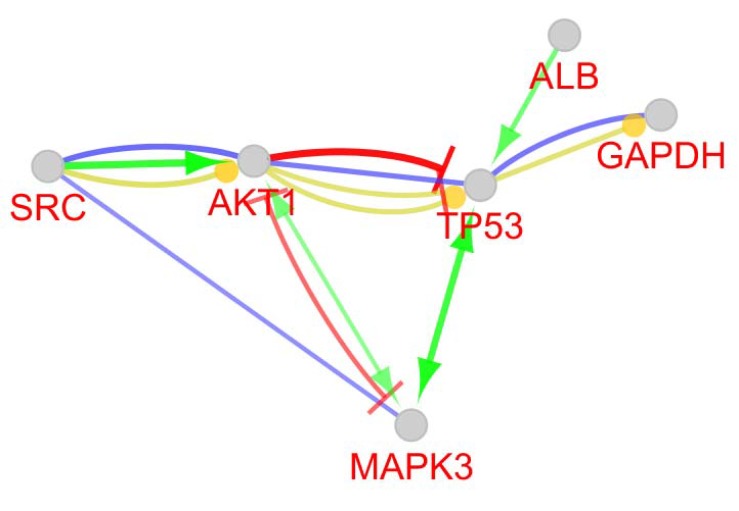
Action map of 6 critical DEGs of colon cancer grades II and III network. Green; activation, red; inhibition, blue; binding, and yellow; expression. Arrow, bar, and round tips are corresponding to activation, inhibition (and down-regulation), and up-regulation respectively

The PPI network included 37 isolated nodes and a main connected component (counting 257 nodes and 5437 edges). Network analysis showed degree distribution is fitted by y = ax ^b^ where a and b are 10.070 and -0.415 respectively. Correlation and R-squared are 0.830 and 0.393 respectively (R-squared is computed on logarithmized values). Based on degree distribution, the network is scale free and it is possible to introduce central nodes (see figure 3). As it is shown in the table 1, numbers of 6 critial nodes based on degree value, betweenness centrality, and closeness centrality are determined. Action network of 6 critical DEGs including expression, activation, inhibition, and binding is illustrated in the figure 4. Gene ontology findings including 34 biological terms related to the 6 critical nodes are presented in the table 2. All terms except HIF-1 signaling pathway, are classified as a one cluster. MAPK3, AKT1, SRC, TP53, GAPDH, and ALB are involved in 34, 32, 21, 17, 1, and zero biological terms respectively.

## Discussion

Colon cancer is divided into 4 grades and treatment in advanced cases has many problems, sometimes is not successful (19). Therefore, researchers are looking for ways to prevent or diagnose the disease so they can cure it quickly before it's too late. The identification of biomarkers that are clearly indicative of this disease could help to diagnose early stages of disease, but the recognition of these biomarkers is definitely not yet possible. The Researchers are looking for ways to find these biomarkers. Different cellular, molecular and pathological methods have been used to identify biomarkers. Identification of genome and proteome of colon cancer cells by conventional laboratory methods and comparing them with each other using database and bioinformatics software has attracted the attention of researchers. Researchers are hoping to find major biomarkers for colon cancer with these new methods. This study also attempts to identify important genes involved in the colon cancer grade II compare to grade III. As the results show MAPK3, AKT1, SRC, TP53, GAPDH, & ALB are the genes involved in colon cancer grade II & III. These genes are in the order of precedence in function. The functional relationship between these genes is shown in Fig 4, it evident mutual overlap and increase the activity of TP53 and MAK3P on each another. On the other hand, AKT1 reduces TP53 activity and increases the activity of MAPK3.The activity of GADPH on other genes was not apparent. SRC also stimulates and increases AKT1 activity. It's best to start with TP53 function in the cell. TP53 is a gene that interferes with cell cycle activities, and is a tumor suppressor. When a tumor suppressor gene mutates, the proliferation of the cells becomes uncontrollable. The most mutated gene in cancer patients is TP53. Disturbance in the regulation of the TP53 tumor suppressor gene is one of the most common events in colorectal cancer stimulation with metastatic aspect of colon cancer and reactivation of TP53 gene may be a suitable suggestion for CC treatment (20). p53 can activate the mitochondrial (intrinsic) and the death-receptor-induced (extrinsic) apoptotic pathways during cell stress (21). *MAPK3* were uniquely associated with *TP53* mutations (22). This stance confirms our findings regarding the interaction between P53 and MAPK3. Researchers have suggested the impact of metformin on MAPK3 for cancer patients (23). It should be noted that the usefulness of MAPK3/1 expression as a biomarker for sensitivity to drugs as metformin is uncertain by others (24). The possible interaction between the AMPK and MAPK3/1 pathways are potential targets for cancer treatment and prevention(19). AKT1gene provides AKT1 kinase protein which plays critical role in cell functions as proliferation and differentiation, cell survival and apoptosis (25). It should be noted that AKT1 is a gene belongs to the group of oncogenes cause the normal cells to become cancerous in mutations (26). Our results showed that AKT1 was reduced TP53 and MAPK3 activity. Due to the role of TP53 as a Tumor suppressor, the AKT1 function on TP53 leads to increase tumor cell proliferation in cancer cells. This is apparently consistent with our result*s.* Therefore, we can evaluate the cycle between the positive effect of SRC on AKT1 and the negative effect of AKT1 on TP53. Eighty percent of patients with colon cancer overexpress SRC in tumor tissue but SRC therapy may causes weaken immune responses (27). It should be noted that the strong immune system is needed to suppress the cancer cells and suppression of the SRC gene as well as having a strong immune system could be effective in treating colon cancer. On the other hand, the interaction between TP53 and MAPK3 should not be forgotten as mentioned above. According to our results which is in line with the other researchers, if it is possible to adjust the SRC activity to AKT1, with a pharmacological mechanism, the TP53 malfunction for colon do not prolong infinite differentiation. In another way if the TP53 functions in colon cancer cells reinforced, it may cause cancer cell apoptosis. Other researchers suggest drugs for target therapy as *Regorafenib *(*Stivarga*) which is a kinase inhibitor. Blocking of kinase proteins could prevent tumor growth and angiogenesis in cancers (28). We suggest a pathway to regulate, not inhibit the effect SCR protein kinase in colon cancer treatment according to the suggestion of other scholars (29, 30). TP53 gene and p53 protein regulation in target therapy is also a favorite of researchers (31) as mentioned in the results of this research.

In conclusion we suggest target therapy to regulate SRC, TP53, AKT1 and MAPK3 could assist treatment of colon cancer and more researches in necessary to design drugs for these purposes.
